# Temporal subtraction CT with nonrigid image registration improves detection of bone metastases by radiologists: results of a large-scale observer study

**DOI:** 10.1038/s41598-021-97607-7

**Published:** 2021-09-16

**Authors:** Koji Onoue, Masahiro Yakami, Mizuho Nishio, Ryo Sakamoto, Gakuto Aoyama, Keita Nakagomi, Yoshio Iizuka, Takeshi Kubo, Yutaka Emoto, Thai Akasaka, Kiyohide Satoh, Hiroyuki Yamamoto, Hiroyoshi Isoda, Kaori Togashi

**Affiliations:** 1 Department of Diagnostic Imaging and Nuclear Medicine, Kyoto University Graduate School of Medicine, 54 Kawahara-cho, Shogoin, Sakyo-ku, Kyoto, Kyoto 606-8507 Japan; 2 Preemptive Medicine and Lifestyle-Related Disease Research Center, Kyoto University Hospital, 53 Kawahara-cho, Shogoin, Sakyo-ku, Kyoto, Kyoto 606-8507 Japan; 3 Medical Products Technology Development Center, R&D Headquarters, Canon Inc., 30-2, Shimomaruko 3-chome, Ohta-ku, Tokyo, 146-8501 Japan; 4 Kyoto College of Medical Science, 1-3 Imakita, Koyamahigashi-cho, Sonobe-cho, Nantan, Kyoto 622-0041 Japan; 5 R&D Headquarters, Canon Inc., 30-2, Shimomaruko 3-chome, Ohta-ku, Tokyo, 146-8501 Japan; 6 Department of Radiology, Kobe City Medical Center General Hospital, 2-1-1 Minatojimaminamimachi, Chuo-ku, Kobe, Hyogo 650-0047 Japan; 7 Department of Radiology, Tenri Hospital, 200 Mishima, Tenri, Nara 632-8552 Japan

**Keywords:** Cancer imaging, Imaging techniques, Software

## Abstract

To determine whether temporal subtraction (TS) CT obtained with non-rigid image registration improves detection of various bone metastases during serial clinical follow-up examinations by numerous radiologists. Six board-certified radiologists retrospectively scrutinized CT images for patients with history of malignancy sequentially. These radiologists selected 50 positive and 50 negative subjects with and without bone metastases, respectively. Furthermore, for each subject, they selected a pair of previous and current CT images satisfying predefined criteria by consensus. Previous images were non-rigidly transformed to match current images and subtracted from current images to automatically generate TS images. Subsequently, 18 radiologists independently interpreted the 100 CT image pairs to identify bone metastases, both without and with TS images, with each interpretation separated from the other by an interval of at least 30 days. Jackknife free-response receiver operating characteristics (JAFROC) analysis was conducted to assess observer performance. Compared with interpretation without TS images, interpretation with TS images was associated with a significantly higher mean figure of merit (0.710 vs. 0.658; JAFROC analysis, P = 0.0027). Mean sensitivity at lesion-based was significantly higher for interpretation with TS compared with that without TS (46.1% vs. 33.9%; P = 0.003). Mean false positive count per subject was also significantly higher for interpretation with TS than for that without TS (0.28 vs. 0.15; P < 0.001). At the subject-based, mean sensitivity was significantly higher for interpretation with TS images than that without TS images (73.2% vs. 65.4%; P = 0.003). There was no significant difference in mean specificity (0.93 vs. 0.95; P = 0.083). TS significantly improved overall performance in the detection of various bone metastases.

## Introduction

A total of 1,762,420 new cancer cases and 660,880 cancer deaths are projected to occur in the United States in 2019^1^. Bone is the third most common organ affected by metastasis^2^. Therefore, early detection of bone metastases is important to improve quality of life for cancer patients and reduce cancer-related mortality.

Bone metastases can cause skeletal-related events (SREs), which are often accompanied by severe pain and quadriplegia^2–5^. SREs are sometimes treated with surgery or radiotherapy, although outcomes are often unsatisfactory^3^. In contrast, several preventative interventions including anticancer chemotherapy, palliative radiotherapy^6,7^, and bone-modifying agents^8,9^, can lead to improved outcomes^10,11^. However, optimal application of such treatments requires early detection of bone metastases.

Early detection is potentially achievable because cancer patients are frequently examined with computed tomography (CT) to detect local recurrence and distant metastasis. Owing to recent advances in CT scanners, bone metastases can be identified as small and faint lesions^12,13^. However, these are disguised by huge amounts of detailed anatomical information that often impedes the ability of radiologists to detect lesions within a reasonable time. In addition, because the CT density of the bone is higher than that of other organs, changes in the CT density due to bone metastases are not clearly depicted on routine CT images. Bone window condition, characterized by wide window width and high window level, is usually applied to CT images for bone viewing. However, its application hinders detection of subtle changes in the CT density of bone.

To exploit the rich information provided by CT, several studies have reported applications of temporal subtraction (TS) based on nonrigid image registration algorithms to CT images^14–21^. Sakamoto et al.^14^ evaluated overall radiologist performance in the detection of newly-developed bone metastases at serial follow-up CT using jackknife free-response receiver operating characteristic (JAFROC) analysis^22,23^, reporting that performance was improved by TS images, although the improvement was not significant. Ueno et al.^15^ observed a significant improvement in performance, although their study focused on osteoblastic metastases of the spine.

**Figure 1:**
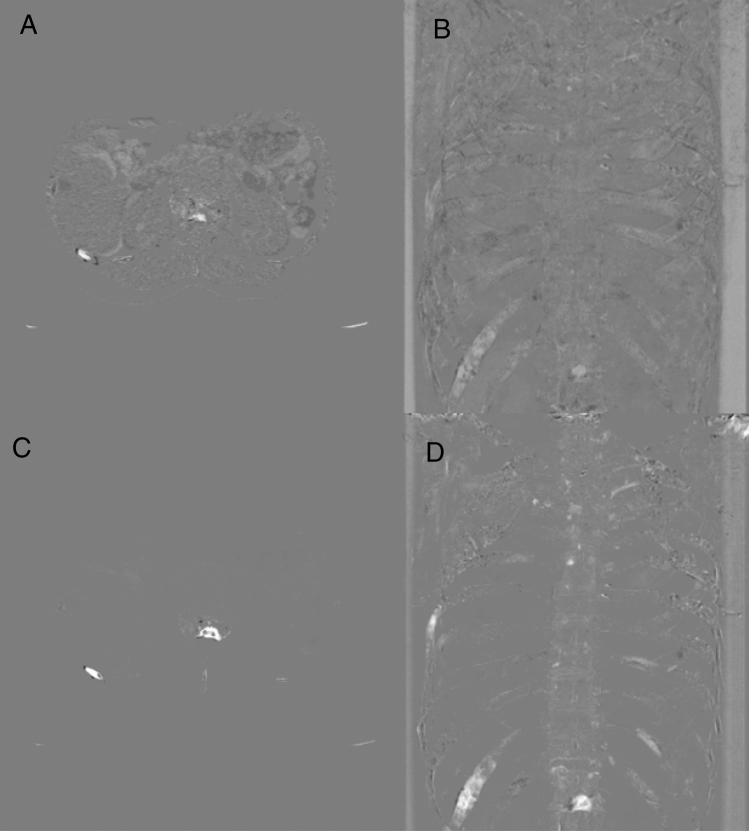
Temporal subtraction (TS) images generated using our TS method and Sakamoto’s TS method. Images are obtained from a 71-year-old female patient with lung cancer who developed multiple osteogenic and mixed bone metastases. (**A**,**B**) An axial image and a projection image generated using Sakamoto’s method, respectively; (**C**,**D**) an axial image a projection image generated using our TS method, respectively.

The purpose of this study is to test the hypothesis that TS images can improve detection of various bone metastases during follow-up CT studies conducted by numerous radiologists with a variety of backgrounds. The TS method used in this study has been substantially improved since Sakamoto’s study^14^ to reduce processing time, noise due to digitizing^24^, and artifacts due to global physiological changes (Fig. 1). This enables clear and efficient visualization of local pathological changes including bone metastases. Major contributions of the current study are summarized as follows:

1. A large-scale observer study was performed for detection of bone metastases. Numbers of radiologists and patients were 18 and 100, respectively.
2. To validate the robustness of detection with TS images, the radiologists with a variety of backgrounds and the patients with various primary tumors and various bone metastases were included. To include various bone metastases, osteoblastic, osteolytic, intertrabecular, and mixed types of newly-developed and preexisting bone metastases at various locations were included.
3. Although the studies of Onoue et al.^18^ and Sakamoto et al.^14^ did not show significant improvement between with and without TS images, the current study shows significant improvement.

## Materials and methods

This retrospective study was approved by the institutional review board (Kyoto University Graduate School and Faculty of Medicine, Ethics Committee), and requirement for informed consent was waived. This study conformed to the Declaration of Helsinki and Ethical Guidelines for Medical and Health Research Involving Human Subjects in Japan (https://www.mhlw.go.jp/file/06-Seisakujouhou-10600000-Daijinkanboukouseikagakuka/0000080278.pdf).

### Subject selection

Six board-certified radiologists (M.Y., M.N., T.K., Y.E., K.O., T.A.; all of these are authors of this paper) with 9–22 years of experience in interpreting CT images selected subjects meeting pre-defined criteria (Supplementary Information A) from a clinical database, sequentially scrutinizing CT images. Briefly, the criteria are as follows. (i) The six board-certified radiologists included subjects with a history of malignancy who were examined with at least three CT studies (previous, current, and future CT). (ii) The subjects had a history of examinations of 18F-fluoro-2-deoxy-d-glucose positron emission tomography and/or bone scintigraphy which was performed for evaluation of bone metastases. (iii) Positive subjects (subjects with bone metastases) had at least one bone metastasis measuring 5 mm or more in diameter. (iv) Negative subjects (subjects without bone metastases) had no bone metastasis.

Supplementary Information B shows the procedure of subject selection. With reference to images from CT and other imaging modalities, they selected 50 positive subjects and 50 negative subjects. Furthermore, they selected a pair of CT images (previous and current CT) for each subject that satisfied predefined criteria (see Supplementary Information A). Negative subjects were selected to match the background characteristics (e.g., age and sex) of positive subjects. The 6 radiologists detected and reviewed all suspicious lesions, and identified lesions over 5 mm or more to create the reference standard. Finally, lesions were determined to be bone metastases with sufficient confidence by consensus. In this procedure, future CT was used for confirming the reference standard.

The three-dimensional region of each bone metastasis was manually segmented on current CT images by consensus. Subject- and lesion-based attributes were investigated as shown in Tables 1 and 2, respectively. Table 1 shows that the CT scan conditions, such as slice thickness and use of contrast media, were different between previous and current CT in some subjects. The following CT scanners (Canon Medical Systems, Otawara, Japan) were used; Aquilion 16 (16-detector row CT), Aquilion 64 (64-detector row CT), Aquilion Prime (80-detector row CT), and Aquilion One (320-detector row CT).

**Table.1:** Subject and image characteristics.

Characteristic	Positive subjects (*n* = 50)	Negative subjects (*n* = 50)
**Subjects**		
**Age (year)**		
< 60	13	11
60 to < 80	34	36
≥ 80	3	3
**Sex**		
Male	30	30
Female	20	20
**Body mass index (kg/cm**^**2**^**)**		
< 18.5 (emaciation)	3	3
18.5 to < 25 (normal)	31	24
≥ 25 (obesity)	8	9
Not available	8	14
**Primary tumor**^a^		
Breast cancer	9	9
Prostate cancer	10	10
Lung cancer	11	11
Other malignancies	27	30
**Images**		
**Scan target of current CT scan**		
Head	2	2
Chest	7	12
Abdomen	2	4
Neck to chest	1	2
Chest to abdomen	30	28
Neck to abdomen	8	2
**Slice thickness (previous/current)**		
≤ 1 mm/≤ 1 mm	41	41
≤ 1 mm/> 1 mm	2	2
> 1 mm/≤ 1 mm	2	2
> 1 mm/> 1 mm	5	5
**Use of intravenous contrast medium (previous/current)**		
+/+	24	16
+/−	4	2
−/ +	2	3
−/−	20	29
**Study interval**		
> 30 days and < 1 year	20	11
≥ 1 year	30	39

*CT* computed tomography.

^a^Multiple options were permitted.

**Table.2:** Lesion characteristics.

Characteristic	*n*
**Size**	
> 5 mm to < 10 mm	34
> 10 mm to < 30 mm	83
≥ 30 mm	43
**Type**	
Osteolytic	33
Osteogenic	83
Intertrabecular	26
Mixed	18
**Location**	
Skull	4
Scapulae	5
Sternal bone, ribs, or claviculae	42
Spine	66
Pelvis	40
Extremities	3
**Newly-developed or preexisting**	
Newly-developed	91
Preexisting	68
Not available (out of scan range at previous CT scan)	1

*CT* computed tomography.

### TS image generation

The process for generating TS images is almost identical to that of Onoue et al.^18^. Previous CT images for each subject were non-rigidly transformed to match current CT images. The non-rigid image transformation was performed fully automatically. Subsequently, transformed previous CT images were subtracted from current CT images to generate TS images using the Intel Xeon E5-1650v4 processor (Clock, 3.50 GHz, number of cores 6; memory, 32 GB). Processing time for TS generation was recorded. Projection images, which were the average of the maximum and minimum intensity projections of TS images, were also generated to enable observers to immediately grasp osseous temporal changes across the whole area.

### Observer enrollment

This experiment was a fully crossed multi-observer multi-subject study. Based on Sakamoto’s study^14^ and according to a sample size calculation conducted with freely available JAFROC software (Rjafroc version 0.1.1.; https://github.com/dpc10ster/RJafroc)^22,23^, 18 radiologists satisfying predefined criteria (Supplementary Information C) were enrolled as observers. The six board-certified radiologists (M.Y., M.N., T.K., Y.E., K.O., T.A) were not included in the 18 observers. Observers’ experience and specialties in radiology were recorded. Multiple options could be selected for their specialties.

### Observer study

To reduce memory bias, observers were randomly assigned to two groups of equal size (*n* = 9). One group independently interpreted the image pairs for each subject first without and then with TS images. The other group interpreted the image pairs first with and then without TS images. The interval between two sessions without and with TS for each observer was more than 30 days. Moreover, the order of subjects was randomized for each observer.

**Figure 2:**
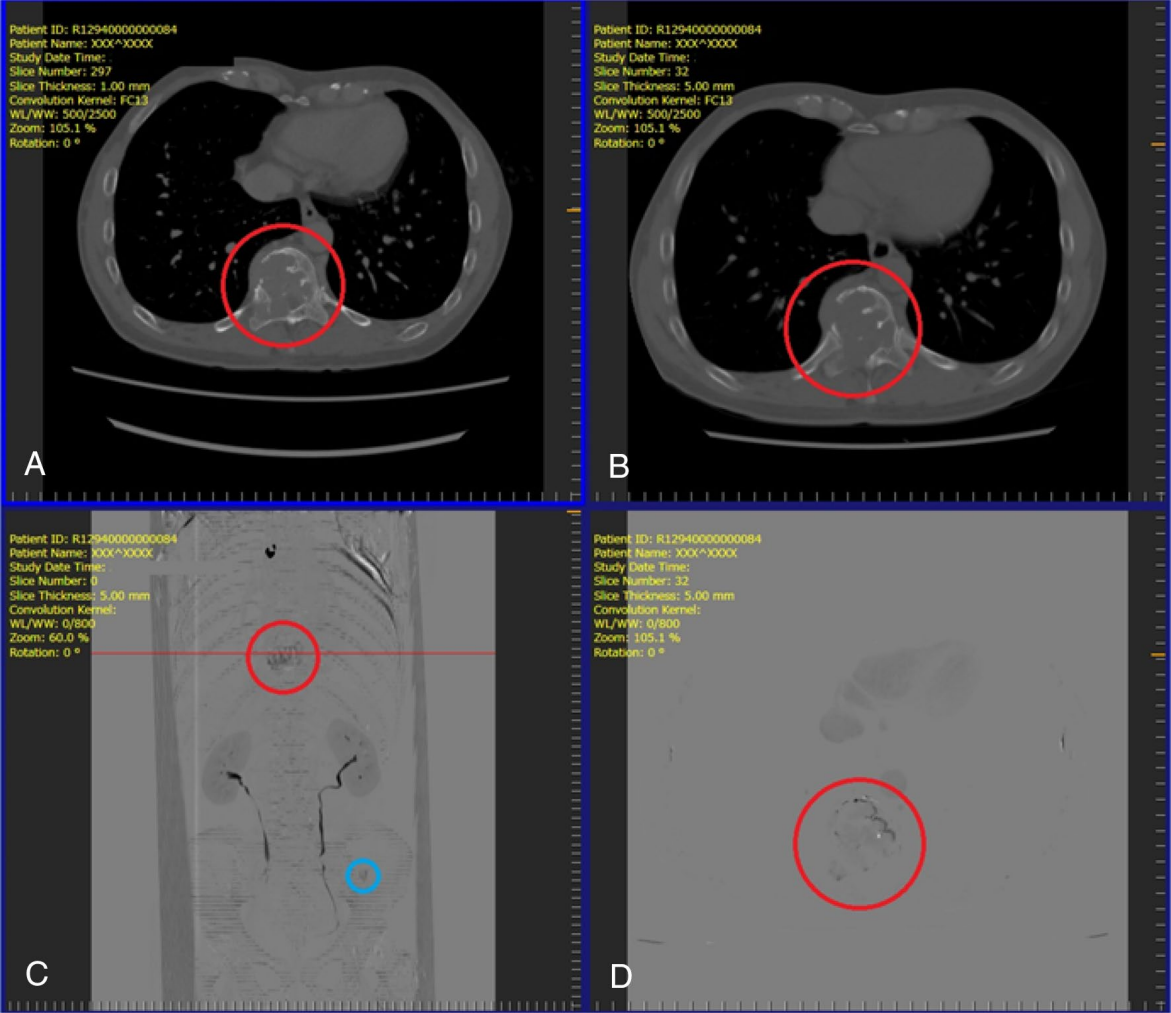
Screenshot of the image viewer for the observer study. (**A**) previous computed tomography (CT) image (upper left); (**B**) current CT image (upper right); (**C**) projection of temporal subtraction (TS) images (lower left); (**D**) TS image (lower right). When an observer clicks on a suspicious lesion, the dialog box appears to rate its likelihood (low to high) of being a bone metastasis. These representative images are obtained from a 55-year-old male patient with renal cell carcinoma who developed two osteolytic metastases in a thoracic vertebra (red circle) and the left iliac bone (blue circle). Both metastases are clearly visualized.

Observers used a medical monitor (Radiforce RX440, EIZO) and a dedicated image viewer (Fig. 2) with multi-planar reconstruction and window level/width modification functions to view CT and TS images. To control practice effects, observers were trained to use the viewer with training data of ten subjects prior to the actual study. Observers were blinded to all clinical data except the age and sex of each subject and the interval between previous and current studies.

Observers were asked to mark the location of any suspicious lesions measuring 5 mm or more on current images and to rate the percentage likelihood of bone metastasis. The interpretation time for each subject was automatically recorded by the viewer excluding the time for rating. After interpretation of each subject, observers were asked to subjectively rate on a five-point scale the confidence level for their interpretation (1, very low; 2, low; 3, moderate; 4, high; 5, very high) and the usefulness of TS images (1, useless; 2, not very useful; 3, somewhat useful; 4, very useful; 5, extremely useful).

After completion of all assessments, the marked locations of lesions were compared against the reference standard for lesion identification. A lesion with a likelihood rating of 51% or higher was considered positive in lesion-based analyses. A subject with at least one positive lesion was considered positive in subject-based analyses. TS images were considered beneficial for identifying lesions where at least one observer could correctly identify and positively rate only with TS images. Meanwhile, they were deemed detrimental to identifying lesions where at least one observer could correctly identify and positively rate only without TS images. All false positives were further reviewed by the six radiologists.

### Statistical analyses

JAFROC analysis^22,23^ was conducted with JAFROC software with random-observers-and-random-subjects models and the figure of merit (FOM) was calculated to evaluate overall observer performance. Sensitivity at lesion-based, false positive count (FPC) per subject, sensitivity and specificity at subject-based, interpretation time, and confidence levels were compared between sessions (with TS images vs. without TS images) with the Wilcoxon signed rank test. SAS (Version 9.4, SAS Institute, Cary, North Carolina) was used for statistical analyses, and *P* < 0.05 was considered to indicate a significant difference.

## Results

### Subject characteristics

Positive subjects (with bone metastases) and negative subjects (without bone metastases), and their image pairs were successfully selected based on the predefined criteria (Supplementary Information A). Subjects included 60 men and 40 women who underwent the latest CT scans between 13 March and 31 July 2017 at the time of subject enrollment. The age of subjects ranged from 14 to 86 years at the time of their current CT scan. For these patients, previous CT scans had been conducted between 12 October 2007 and 6 March 2017 while current CT scans had been conducted between 29 October 2009 and 17 May 2017, respectively. The interval between previous and current CT scans ranged from 31 to 1973 days. Some image pairs had inconsistencies in intravenous contrast medium, slice thickness, and scan area between previous and current scans. Detailed characteristics for the 100 subjects are shown in Table 1 and Supplementary Information D. In total, the reference standard consisted of 160 bone metastases. Their detailed characteristics are shown in Table 2 and Supplementary Information E.

TS images were generated for the image pairs of the 100 subjects. The mean processing time per image pair was 973 s (range 322–2310, standard deviation 405). TS images were not generated for one metastasis because it was out of the scan area of the previous CT image.

### Observer characteristics

All 18 enrolled observers were board-certified radiologists, with the following specialties in radiology: general radiology (*n* = 4), nuclear medicine (*n* = 2), neuroradiology (*n* = 2), cardiovascular radiology (*n* = 1), respiratory radiology (*n* = 2), upper abdominal radiology (*n* = 6), gastrointestinal radiology (*n* = 2), and urological radiology (*n* = 2). They had 10–36 years of experience in the interpretation of CT images. In clinical practice, they interpreted 3000 to 10,000 CT examinations each year. Two radiologists had previously used computer-aided diagnosis system. None had previously used TS-CT.

### Image interpretation

**Figure 3:**
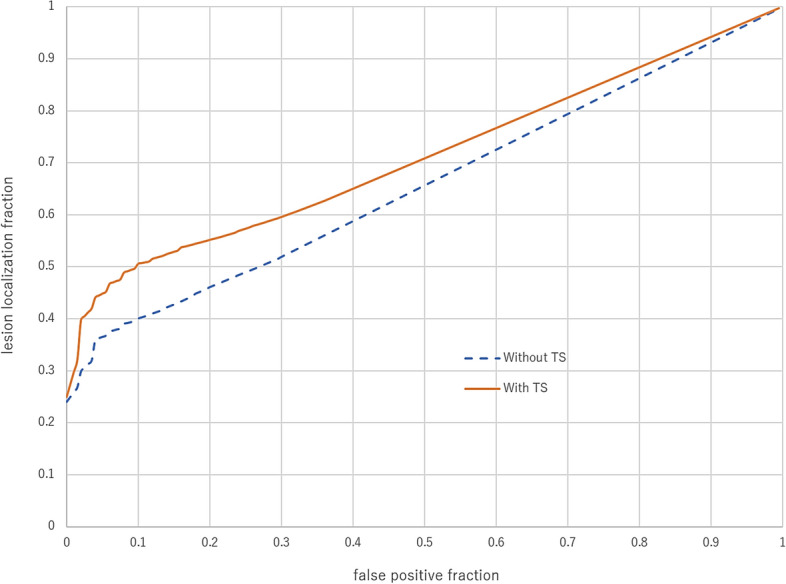
Average free-response receiver operating characteristic curves without (dotted blue line) and with (red solid line) temporal subtraction (TS) images. Radiologist performance significantly increased with the assessment with TS compared with assessment without TS.

**Figure 4:**
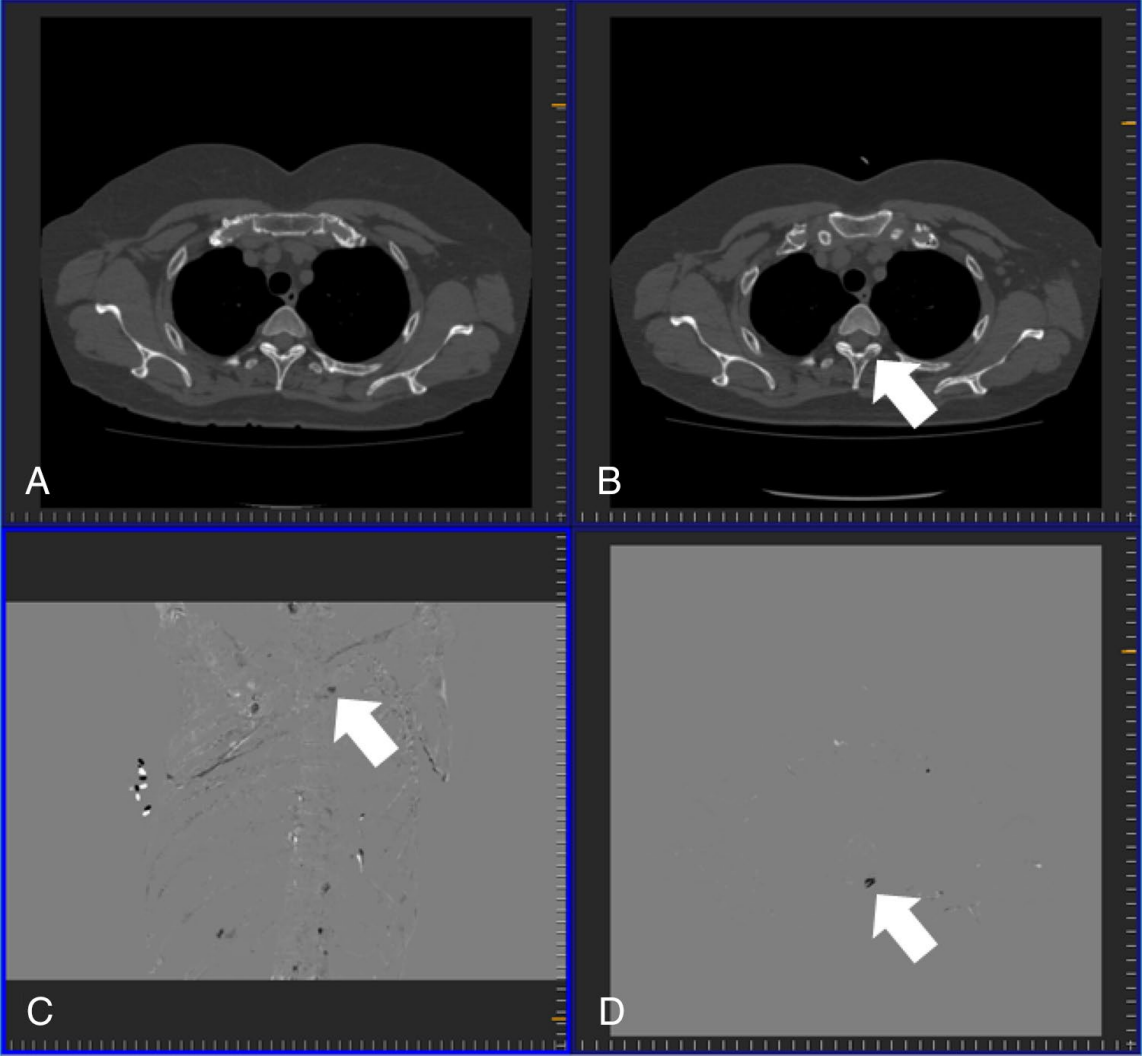
Osteolytic metastasis in the Th3 vertebra (arrow) of a 54-year-old female patient with breast cancer. (**A**) Previous computed tomography (CT) image; (**B**) current CT image; (**C**) temporal subtraction (TS) image; (**D**) projection image of TS images.

**Figure 5:**
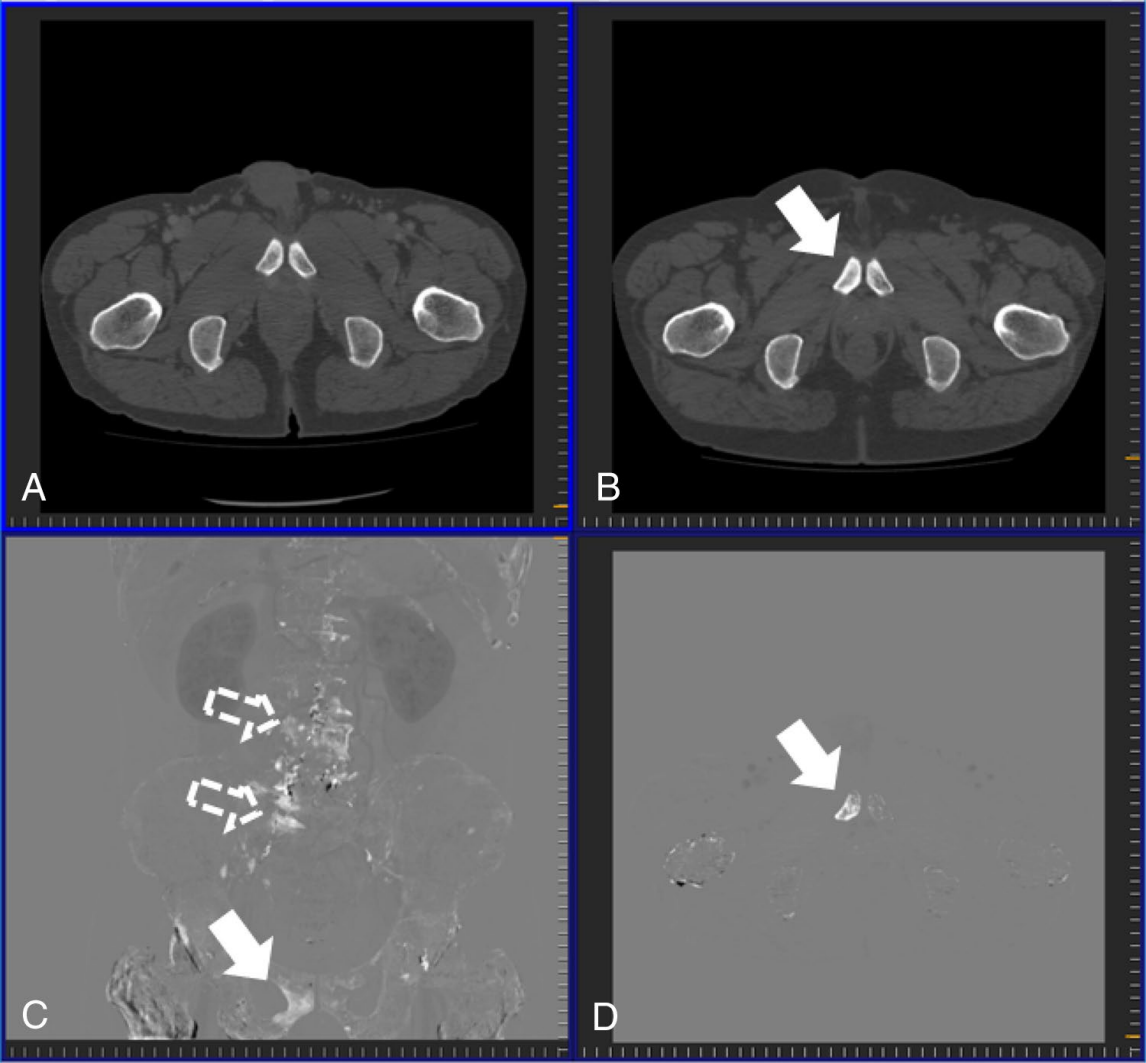
Osteogenic metastasis in the right pubis (arrow) accompanied by degenerative changes (outlined arrow) in a 59-year-old male patient with prostate cancer. (**A**) Previous computed tomography (CT) image; (**B**) current CT image; (**C**) temporal subtraction (TS) image; (**D**) projection image of TS images.

The 18 observers evaluated the 100 image pairs with and without TS. In total, 3600 reading sessions were performed. Figure 3 and Table 3 show the main results for image interpretation. Representative cases are shown in Figs. 4 and 5. Compared with interpretation without TS, TS images were associated with a significant increase in mean FOM from 0.658 to 0.710 (JAFROC analysis, *P* = 0.0027). Mean sensitivity at lesion-based was significantly higher for interpretation with TS compared with that without TS (46.1% [73.8 of 160] vs. 33.9% [54.2 of 160]; *P* = 0.003). Mean FPC per subject was also significantly higher for interpretation with TS than for that without TS (0.28 vs. 0.15; *P* < 0.001).

**Table.3:** Figure of merit, lesion-based sensitivity, false positive count per patient, subject-based sensitivity and specificity, interpretation time, and confidence level without and with TS, and usefulness ratings for observers across all subjects.

Observer	A	B	C	D	E	F	G	H	I	J	K	L	M	N	O	P	Q	R	Average
**Figure of merit**																			
Without TS	0.687	0.638	0.697	0.709	0.516	0.595	0.641	0.699	0.605	0.710	0.710	0.558	0.668	0.599	0.748	0.631	0.754	0.670	0.658 (0.063)
With TS	0.800	0.741	0.785	0.727	0.520	0.659	0.700	0.745	0.582	0.777	0.816	0.613	0.717	0.670	0.739	0.730	0.797	0.653	0.710 (0.078)
**Lesion-based sensitivity**																			
Without TS	0.444	0.375	0.413	0.500	0.231	0.281	0.319	0.406	0.300	0.369	0.438	0.212	0.369	0.225	0.256	0.188	0.338	0.444	0.339 (0.093)
With TS	0.663	0.531	0.594	0.544	0.313	0.331	0.481	0.516	0.394	0.538	0.638	0.338	0.381	0.338	0.381	0.425	0.481	0.438	0.461 (0.109)
**False positive count per patient**																			
Without TS	0.150	0.160	0.100	0.380	0.260	0.080	0.080	0.260	0.230	0.070	0.170	0.100	0.140	0.020	0.040	0.030	0.040	0.320	0.146 (0.106)
With TS	0.420	0.500	0.220	0.310	0.720	0.090	0.420	0.320	0.320	0.230	0.320	0.220	0.090	0.110	0.060	0.080	0.170	0.380	0.277 (0.173)
**Subject-based sensitivity**																			
Without TS	0.800	0.760	0.780	0.740	0.480	0.620	0.700	0.760	0.680	0.680	0.720	0.520	0.680	0.460	0.560	0.460	0.600	0.780	0.654 (0.116)
With TS	0.920	0.760	0.780	0.760	0.620	0.640	0.820	0.820	0.720	0.800	0.760	0.660	0.600	0.640	0.680	0.800	0.620	0.760	0.731 (0.088)
**Specificity**																			
Without TS	0.960	0.920	0.960	0.880	0.840	0.980	0.980	0.960	0.900	1.000	0.980	0.920	0.960	0.980	1.000	1.000	1.000	0.920	0.952 (0.046)
With TS	0.920	0.880	0.960	0.900	0.820	0.980	0.880	0.960	0.740	0.980	0.960	0.940	1.000	0.980	1.000	0.980	0.960	0.920	0.931 (0.068)
**Interpretation time (s)**																			
With TS	242	131	98	261	284	230	125	293	209	158	232	391	273	137	401	255	404	334	248 (95)
Without TS	329	110	125	197	331	183	192	327	184	127	318	211	432	112	372	165	532	195	247 (120)
**Confidence levels**																			
With TS	3	4	3	4	3	4	3	3	3	3	5	3	3	4	3	2	3	3	3
Without TS	4	4	4	3	3	4	4	3	4	3	5	3	3	4	3	3	3	3	3
Usefulness	4	4	5	4	3	4	4	3	4	4	5	3	3	4	3	3.5	3	3	3.75

Averages are mean (standard deviation) or median. Median is used in confidence levels and usefulness. Cells for which result is inferior in a comparison between without and with TS are bold.

*TS* temporal subtraction.

At the subject-based, mean sensitivity was significantly higher for interpretation with TS images than that without TS images (73.2% [36.6 of 50] vs. 65.4% [32.7 of 50]; *P* = 0.003). However, there was no significant difference in mean specificity (0.93 [46.6 of 50] vs. 0.95 [47.6 of 50]; *P* = 0.083) or mean interpretation time (248 s vs. 247 s; *P* = 0.913) between the two sessions.

Median confidence levels ranged from 2 (low) to 5 (very high) for interpretations without TS and from 3 (moderate) to 5 (very high) for those with TS. The median ratings for usefulness of TS images ranged from 3 (somewhat useful) to 5 (very useful), indicating that all observers evaluated TS as useful.

Subjects were divided into subgroups according to the type, location, and preexistence of bone metastases (Table 4). Sensitivity with TS was higher than or equal to that without TS for all subgroups. The gain in sensitivity for interpretation with TS compared with that without TS was small in metastases in the scapulae. Moreover, the gain for metastases in extremities was zero because sensitivity for both interpretations without and with TS were also zero.

**Table.4:** Subgroup results based on metastases characteristics.

Characteristic	Sensitivity without TS	Sensitivity with TS
**Metastasis type**		
Osteolytic	**0.534**	0.613
Osteogenic	**0.301**	0.461
Intertrabecular	**0.024**	0.120
Mixed	**0.611**	0.679
**Metastasis location**		
Skull	**0.583**	0.611
Scapulae	**0.267**	0.289
Sternal bone, ribs or claviculae	**0.245**	0.398
Spine	**0.391**	0.472
Pelvis	**0.361**	0.550
Extremities	0.000	0.000
**Newly-developed or preexisting**		
Newly-developed	**0.314**	0.471
Preexisting	**0.376**	0.454
Not available (out of scan range at previous CT)	0.000	0.000
**Metastasis size**		
> 5 mm to < 10 mm	**0.106**	0.258
> 10 mm to < 30 mm	**0.292**	0.398
≥ 30 mm	**0.612**	0.743

Sensitivity without and with TS for each subgroup of the 160 bone metastases is shown. Cells for which result is inferior in a comparison between without and with TS are bold.

*CT* computed tomography, *TS* temporal subtraction.

### Effects of TS images on metastases detection

Of the 160 metastases, a beneficial effect of TS images was observed for 118 and a detrimental effect was observed for 82. In particular, there were eight notable metastases for which detection was improved by TS images for 10–15 of the 18 observers, while a detrimental effect was observed for 0–1 observer. These metastases comprised not only three small metastases but also five larger ones, measuring 21.8–32.9 mm. These larger lesions were “lost” on current CT images, disguised by commonly-observed degenerative changes and sterically complex structures of the sternum, ribs, or pelvic bones.

In contrast, there were seven notable metastases for which detection was detrimentally affected by TS images for 5–8 observers, while a beneficial effect was observed for 0–2 observers. These metastases resembled commonly-observed benign findings on TS images, especially the projection images, such as degenerative changes of the vertebrae and joints, healing fractures of the ribs and pelvic bones, and subtraction artifacts around the scapulae.

The review of false positive marks without and with TS images identified 161 and 212 bone lesions, respectively. In most of the false positives (n = 130 without TS and 148 with TS), the number of observers who marked was one, while the number was 5 to 15 in some lesions (n = 7 without TS and 23 with TS). It is speculated that these lesions represent degenerative changes (*n* = 4 without TS and 10 with TS), healing fractures (*n* = 1 without TS and 6 with TS), post-operative changes (*n* = 1 without TS and 1 with TS), and other benign bone lesions (*n* = 1 without TS and 6 with TS) such as bone islands.

## Discussion

This study investigating the effects of TS on bone metastases detection in CT images indicated that TS images could be made available at follow-up CT without any extra physical burden on patients. Moreover, TS images significantly improved overall performance in detection of various types of bone metastases at various locations by radiologists without additional interpretation time. This study recruited a relatively large number of radiologists to assess CT images from a large number of subjects. Furthermore, considering the frequency of CT scans in oncology patients, we believe that our TS method could bring considerable benefit to clinical diagnostic imaging.

This is the first study to report a significant improvement in overall radiologist performance at detecting various types of newly-developed and preexisting bone metastases at various locations by using TS images. Table 4 suggests that TS was beneficial for all types of bone metastases unlike ^18^F-fluoro-2-deoxy-d-glucose positron emission tomography or bone scintigraphy, which are reported to only have benefits for specific metastases^25–27^. Moreover, TS retains the advantages of CT, which has finer resolution and is more frequently performed in oncology patients than other imaging modalities. All these advantages are essential for earlier detection of bone metastases.

TS method is clinically applicable because our study evaluated TS images without excluding subjects for inconsistencies between previous and current CT images in posture, breathing depth, and other study attributes (Table 1), which are inevitable with real-world application. Furthermore, these results were obtained with the 18 radiologists who have various backgrounds and no previous experience of TS for bone metastasis detection. Moreover, TS is likely to be accepted by radiologists based on their usefulness ratings. As such, clinical application of TS could enable early detection of bone metastases, reducing SRE and cancer-related mortality and improving quality of life of cancer patients.

There were some detrimental effects of TS on detection, which can presumably be attributed to conspicuous visualization of commonly-observed degenerative and traumatic changes, premature judgment of such changes or bone metastases on TS images, and abbreviated observation of CT images based on this judgment. To minimize these effects, radiologists should be educated about TS. Some visualization aids might also be helpful to minimize such effects, including image fusion and synchronized scrolling to assist radiologists in exploiting both CT and TS information.

It was observed that sensitivity for intertrabecular metastases was lower than that for other types even with TS. Although TS improved sensitivity, the improvement was smaller presumably due to a smaller density change. To increase the advantage gained with TS images for such metastases, computer-aided detection might be developed.

By its nature, the TS method used here exploits follow-up CT images and requires prior images, which are unavailable at initial imaging assessments. In such situations, another modality should also be considered because some cancer patients already have bone metastases at initial diagnosis^28–30^. However, follow-up evaluations, as well as detection of bone metastases, are important for their management, including the prevention of SRE. Based on Table 4, TS appears to assist radiologists in identifying both preexisting and newly-developed bone metastases. Follow-up evaluation of bone metastases with CT is generally considered difficult in some cases^31^. Further research is therefore required to investigate the use of TS for follow-up evaluations.

Although the processing time in this study was much shorter than that of Sakamoto’s study^14^, it would be preferable to further shorten it for clinical application of TS, especially in emergency CT assessments for SRE. According to preliminary results using in-house software, processing time can be reasonably expected to be reduced to less than 10 min with the use of a graphics processing unit.

There were several previous studies for investigating the usefulness of TS for detection of bone metastases^14–16,18,21^. To the best of our knowledge, the current study was the first to show that TS was useful for detecting bone metastases even when inconsistent CT sets (such as slice thickness) were included for generating TS.

There were several limitations to this study. First, despite repeated scrutinization of CT images by the 6 board-certified radiologists, reference to all available images including those obtained after current images, and determination with sufficient confidence by consensus, the definition of the reference standard might be incomplete because any use of clinical information other than images was not accepted by the Japanese regulatory body (Pharmaceuticals and Medical Device Agency). This study was conducted as a clinical performance test for which the results were to be submitted to the body for approval of TS for clinical use^32^. Although TS images would have considerably assisted the definition of the reference standard, they were not referred to for the definition. Second, TS effects were not sufficiently evaluated for metastases in the skull, scapulae, and extremities due to the small number of subjects with these metastases. All three metastases in extremities happened to be too difficult to detect and differentiate with CT without reference to other modality images. Therefore, further studies focusing on specific types of metastases are also required. Third, the effect of bone metastasis therapy on detectability with TS was not examined in the current study. The therapy can change CT density of bone metastases^33,34^. Therefore, detectability with TS may be changed with the bone metastasis therapy. Because the access of medical records was severely restricted in performing the current study^32^, we could not examine the effect of bone metastasis therapy on detectability with TS.

In conclusion, TS images obtained from serial CT scans using nonrigid image registration significantly improved radiologist performance in the detection of bone metastases.

## Supplementary Information

Supplementary Information 1.

Supplementary Information 2.

Supplementary Information 3.

## Abbreviations

CT: Computed tomography
FOM: Figure of merit
JAFROC: Jackknife free-response receiver operating characteristic
SRE: Skeletal-related events
TS: Temporal subtraction
FPC: False positive count
