# Supplementary material for: Temporal subtraction CT with nonrigid image registration improves detection of bone metastases by radiologists: results of a large-scale observer study
Source: Sci Rep. 2021 Sep 16;11:18422. doi: 10.1038/s41598-021-97607-7 (PMC8446090; doi:10.1038/s41598-021-97607-7)
Supplement: Supplementary file 1 — Supplementary Information 1. [file 41598_2021_97607_MOESM1_ESM.docx]

**Title Page**

**Full Title**

Temporal Subtraction CT with Nonrigid Image Registration Improves Detection of Bone Metastases by Radiologists: Results of a Large-scale Observer Study

**Authors**

Koji Onoue, MD, PhD, Masahiro Yakami, MD, PhD, Mizuho Nishio, MD, PhD, Ryo Sakamoto, MD, PhD, Gakuto Aoyama, MS, Keita Nakagomi, MS, Yoshio Iizuka, MS, Takeshi Kubo, MD, PhD, Yutaka Emoto, MD, PhD, Thai Akasaka MD, PhD, Kiyohide Satoh, PhD, Hiroyuki Yamamoto, PhD, Hiroyoshi Isoda, MD, PhD, Kaori Togashi, MD, PhD

# Supplementary material A: Predefined Criteria for Subject Selection

Inclusion Criteria for Patients

- A patient has a history of malignancy.
- Images are available from at least two CT studies (previous and current CT) that were obtained with a CT scanner at our institution for diagnostic imaging from 1 February 2007 to 31 July 2017. In addition, future CT was available after previous and current CT.
- A patient has a history of examinations of ^18^F-fluoro-2-deoxy-D-glucose positron emission tomography (PET) and/or bone scintigraphy which was performed for evaluation of bone metastases.

Exclusion criteria for Patients

- A patient refused the usage of his/her image data.

Inclusion Criteria for CT Studies

- CT Images are axial images obtained with a CT scanner at our institution for diagnostic imaging.
- CT Images were reconstructed with a soft-tissue kernel, with slice thickness of 5 mm or less and pixel size of 2 mm or less.
- A patient was scanned in a supine position.
- Non-contrast-enhanced CT images or non-early-phase contrast-enhanced CT images (80 seconds or more passed after administration of contrast medium).
- All bones, except the bones of the extremities or scapulae, were included within the field of view at CT scan slice.
- The size of the field of view was 300 mm or more, or the images were obtained for head CT study.
- Cephalocaudal length of CT scan was 300 mm or more, or the images were obtained for head CT study.

Exclusion Criteria for CT Studies

- Images are obtained with post-processing techniques such as multiplanar reconstruction or volume rendering.
- Images were obtained with a PET/CT scanner or a radiotherapy planning system.
- Images have severe artifacts.
- More than 10 bone metastases are depicted.

Inclusion Criteria for Current CT Study

- With reference to available images of other modalities for the patient, radiologists can determine with sufficient confidence whether each suspicious bone lesion in the images of the current CT study is bone metastasis or not.
- Appropriate images of at least one previous CT study are available that satisfy the criteria for previous CT study.

Necessary Conditions for Radiologists to Interpret Current CT Images with Sufficient Confidence

- Images from at least one further CT scan are available that were obtained more than six months after the current CT study and for which the image area covers that of the current CT images.

Inclusion Criteria for Current Images in Positive Subjects

- At least one bone metastasis measuring 5 mm or more in diameter is depicted.
- No more than ten bone metastases are depicted.

Inclusion Criteria for Current CT Images in Negative Subjects

- No bone metastasis is depicted.

Inclusion Criteria for Previous CT Images

- Previous CT study was performed more than one month earlier than the current CT study at our institution.
- Scan range of the previous CT study overlaps with more than half of that of the current CT study.

Preferences for Previous CT Images (The six radiologists selected previous CT study by consensus with consideration of the following preferences.)

- Large overlap in previous and current image areas.
- Interval between previous and current CT studies is close to five years.
- CT scan conditions, such as posture, depth of breathing, and use of intravenous contrast medium, were similar between previous and current CT studies.
- Little or no change due to surgery is found between previous and current CT images.

## Supplementary material B: Procedure of Subject Selection

Patient selection was performed by six radiologists for training data and observer study. The six radiologists carefully selected the subjects who met Predefined Criteria for Subject Selection shown in Supplementary material A in order to avoid selection bias.

In the current study, it is necessary that the attributes of patients and bone metastases must be approximately the same between positive and negative subjects. In addition, bone metastases at head and limbs must be included in the positive subjects.

The six radiologists sequentially evaluated the CT images of 1030 subjects to check whether or not the subject met Predefined Criteria for Subject Selection. From the 1030 subjects, 54 positive and 762 negative subjects met the criteria.

The other 214 subjects were excluded because of the following reasons:

- No CT was available which was performed six months or later after the current CT study (N = 201).
- The number of bone metastases was more than 10 (N = 1).
- Although ^18^F-fluoro-2-deoxy-D-glucose PET and/or bone scintigraphy was performed, the purpose of the examination was not evaluation of bone metastases (N = 5).
- Even with reference to available images of other modalities for the subjects, the 6 radiologists could not determine the existence or number of bone metastasis (N = 3).
- Others (N = 4).

From the 54 positive and 762 negative subjects, a total of 100 subjects (50 positives and 50 negatives) were selected in the order of CT examination date. Then, the attributes of subjects and bone metastases were evaluated.

At least, two of the six radiologists visually evaluated three sets of CT images (previous, current, and future CT) and determined the ground truth of bone metastases in these 100 subjects. If possible, bone scintigraphy with SPECT and FDG-PET/CT were used for determining the ground truth. If the results of two radiologists were inconsistent, one radiologist selected from the other four radiologists determined the ground truth by consensus of the three radiologists.

At this time, since there were no positive subjects of bone metastases at head, head CT images of additional 133 subjects were evaluated by the six radiologists. Then, the six radiologists selected 2 positive subjects of bone metastases at head from the 133 subjects. As a result, 56 positive and 762 negative subjects were selected for the training data and observer study.

For the observer study, 50 positive and 50 negative subjects, which included 2 positive subjects of bone metastases at head, were selected from the 56 positive and 762 negative subjects in the order of CT examination date. Then, attributes of the 100 subjects were checked. After the selection of 100 subjects, 10 subjects were selected for the training data.

## Supplementary material C:

## Predefined Criteria for Observers

Inclusion Criteria

- Three or more years of experience in the interpretation of CT images.
- Interpretation of 1,000 or more CT examinations per year.

Exclusion Criteria

- Authors of the study.
- Conflict-of-interest concerning this study.
